# Prevalence and associated factors of primary dysmenorrhea among women in sub-Saharan Africa: a systematic review and meta-analysis.

**DOI:** 10.1186/s12905-026-04379-1

**Published:** 2026-03-05

**Authors:** Bekan Gudata Gindaba, Takele Mitiku Tesema, Firafan Shuma Teka, Gemechis Ifa Wakjira, Misgana Tesgera Abdisa, Mulugeta Lemma Neggasa, Tesfaye Abera Gudeta

**Affiliations:** 1 Department of Midwifery, Wollega University, Nekemte, Ethiopia; 2 Department of Nursing, Wollega University, Nekemte, Ethiopia; 3 Department of Clinical Pharmacy and Pharmacy Practice, Institute of Health Sciences, Dambi Dollo University, Oromia, Ethiopia; 4 College of Health Science, Assosa University, Assosa, Ethiopia; 5 Department of Nursing, College of Health Sciences, Mattu University, Mattu, Ethiopia

**Keywords:** Primary dysmenorrhea, Prevalence, Associated factors, Sub-Saharan Africa, Systematic review, Meta-analysis

## Abstract

**Background:**

Primary dysmenorrhea, painful menstruation without underlying pelvic pathology, is a prevalent gynecological issue affecting adolescents and young women. Despite its high burden in Sub-Saharan Africa, regional estimates of prevalence and associated factors remain limited. This systematic review and meta-analysis aimed to synthesize evidence on the prevalence and determinants of primary dysmenorrhea among women in Sub-Saharan Africa.

**Methods:**

A comprehensive search of PubMed, Scopus, HINARI, Web of Science, Google Scholar, and university repositories was conducted for studies published between 2008 and 2025. Cross-sectional studies reporting prevalence and/or associated factors of primary dysmenorrhea among SSA women were included. Data extraction and quality appraisal followed the Joanna Briggs Institute tools. Pooled prevalence and associations were estimated using random- or fixed-effects models in STATA 17, with heterogeneity assessed using I², Cochrane Q-test, and publication bias assessed using funnel plots, and Egger’s test.

**Results:**

Sixty-five studies with 28,813 participants from 12 Sub-Saharan African countries were analyzed. The pooled prevalence of primary dysmenorrhea was 73.49% (95% CI: 70.95–76.03). Significant predictors included family history of dysmenorrhea (OR = 5.20; 95% CI: 3.28–7.12), irregular menstrual cycles (OR = 2.57; 95% CI: 1.67–3.48), and short cycle length (< 21 days; OR = 3.31; 95% CI: 1.88–5.83), whereas sexual intercourse was associated with lower odds (OR = 0.39; 95% CI: 0.11–0.68). Subgroup analyses indicated variation across countries, and sensitivity analyses confirmed robustness.

**Conclusion:**

Primary dysmenorrhea affects nearly three-quarters of women in Sub-Saharan Africa and is influenced by family history of dysmenorrhea, irregular menstrual cycles, short cycle length, and sexual intercourse.

**Supplementary Information:**

The online version contains supplementary material available at 10.1186/s12905-026-04379-1.

## Introduction

Dysmenorrhea, defined as painful menstruation, is one of the most frequent gynecological diseases affecting teenagers and young women worldwide [1]. It is categorized into primary dysmenorrhea, which occurs in the absence of recognizable pelvic pathology, and secondary dysmenorrhea, which is associated with underlying diseases such as endometriosis or pelvic inflammatory disease [2]. Primary dysmenorrhea often develops within the first few years after menarche, and it is mainly related to excessive production of uterine prostaglandins, leading to increased uterine contractions, diminished uterine blood flow, and ischemic discomfort [3, 4].

Globally, the prevalence of primary dysmenorrhea among young women ranges from 45% to 95%, with major variation across areas and populations [5]. Evidence consistently reveals that teenagers and young women face the highest burden, often having moderate to severe pain that interferes with everyday activities, academic performance, and psychosocial well-being [6–8]. Despite its high prevalence, primary dysmenorrhea remains under-recognized and inadequately managed, particularly in low- and middle-income countries [9].

In SSA, menstrual health issues, including dysmenorrhea, are compounded by sociocultural taboos, limited access to reproductive health services, and inadequate pain management strategies [10, 11]. Available primary studies from SSA report a high prevalence of dysmenorrhea among school and university students, frequently exceeding 70% in Ethiopia [12], 90% in Uganda [13], 75% in Zimbabwe [14], and more in other countries. The condition has been associated with school absenteeism, reduced academic engagement, diminished quality of life, and increased reliance on self-medication or ineffective traditional remedies [12, 14, 15]. However, menstrual pain is often normalized, resulting in delayed care-seeking and underreporting [15].

Several factors have been implicated in the occurrence and severity of primary dysmenorrhea, including younger age, early age at menarche, irregular menstrual cycles, longer duration of menstrual flow, family history of dysmenorrhea, psychological stress, and lifestyle-related factors such as caffeine intake and poor dietary habits [4, 12, 14, 16]. Although these associations have been explored in individual studies across different SSA countries, findings are inconsistent and fragmented, limiting their applicability for policy formulation and clinical decision-making.

To date, there is no comprehensive systematic review and meta-analysis that synthesizes evidence on the prevalence and associated factors of primary dysmenorrhea among young women in sub-Saharan Africa. The absence of pooled regional estimates hinders a clear understanding of the problem’s magnitude and the identification of key modifiable risk factors. Therefore, this systematic review and meta-analysis aim to estimate the pooled prevalence of primary dysmenorrhea and to identify its associated factors among young women in SSA. The findings are expected to provide robust evidence to inform reproductive health programs, guide targeted interventions, and support the integration of menstrual pain management into adolescent and young women’s health policies in the region.

## Methods

### Protocol and registration

The PRISMA 2020 standards for reporting systematic reviews and meta-analyses were followed in the formulation of the methodology for this systematic review. The four stages of the PRISMA flow diagram, which are described in full in the findings section and show the evolution from initial record identification to final study inclusion, were followed in the study selection procedure [17]. Every pertinent item on the PRISMA checklist was included (**Additional file 1**). Additionally, the review procedure was registered under the registration number CRD420251126040 with PROSPERO, the international prospective register of systematic reviews.

### Inclusion and exclusion criteria

For inclusion, the studies with case-control, cohort, and cross-sectional designs were considered. Unfortunately, the papers we found were all cross-sectional studies. Eligible papers were required to report the prevalence of primary dysmenorrhea or dysmenorrhea and/or at least one risk factor for primary dysmenorrhea or dysmenorrhea among sub-Saharan Africa women. Only studies published in English were included. However, studies that did not focus on the pertinent outcomes, duplicate studies, anonymous reports, and those without abstracts were excluded. Furthermore, after at least two email contacts with the corresponding author, studies that did not provide results for both the exposed and non-exposed groups were excluded because there was no hard data to pull from this research.

### Searching strategy

We searched a wide range of gray literature sources and publications from international databases, including PubMed, Google Scholar, HINARI, Scopus, Google, MEDLINE, Cochrane Library, and Web of Science, as well as university online repositories. The key search terms for the study questions were created using the PICO methodology. These were (Dysmenorrhea) OR (primary dysmenorrhea)) OR (menstrual pain)) AND (Prevalence)) OR (epidemiology)) OR (magnitude)) OR (frequency)) OR (incidence)) AND (Risk Factors)) OR (associated factor)) OR (determinant)) OR (predictor)) AND (Sub-Saharan Africa Countries). The search strategy was employed from September 1, 2025, to October 1, 2025 (Additional file 2).

### Data extraction

Once all relevant research was collected from the databases, the articles were imported into EndNote version 20 and then transferred to a Microsoft Excel spreadsheet to remove duplication. Two reviewers (BG and TM) independently collected significant data using a standardized data extraction technique developed by the Joanna Briggs Institute (JBI) [18]. To resolve disagreements amongst the reviewers, a third reviewer (TA) was consulted. The extracted data included the author’s name, sample size, year of publication, study location, prevalence, and factors associated with primary dysmenorrhea.

### Quality assessment

The methodological quality of included studies was assessed using the Joanna Briggs Institute (JBI) critical appraisal tools. Studies reporting prevalence outcomes were appraised using the JBI checklist for prevalence studies, which includes an appropriate sampling frame, adequate sampling, a sufficient sample size, a detailed setting description, and analysis with sufficient coverage. The checklist also requires a valid method to identify the condition, a reliable measurement, an appropriate statistical analysis, and an adequate response rate. For studies reporting associated factors, the checklist requires clear inclusion criteria, detailed study subjects and setting, valid and reliable measurement of exposure, objectives, and standard criteria for measuring the condition, identification of confounding factors, strategies to address confounding variables, valid and reliable measurement of outcomes, and an appropriate statistical analysis. Both prevalence and associated factors were appraised using the JBI analytical cross-sectional studies checklist. Studies were considered to have a low risk of bias if they met at least 50% of the quality assessment criteria (Additional files 3 and 4).

### Outcome of measurements

#### Primary dysmenorrhea

an article that fits the inclusion criteria with primary dysmenorrhea and/or dysmenorrhea that is operationalized as pain during the menstrual cycle without pelvic pathology.

### Data analysis

STATA version 17 was used to analyze the extracted data for both prevalence and associated factors. Moreover, publication bias was assessed using a funnel plot and Egger’s regression test [19]. The Cochrane Q-test and the I² statistic were used to evaluate heterogeneity among the included studies [20]. The pooled estimates were computed using a weighted, inverse-variance-based random-effects model [21]. The study countries carried out subgroup analysis. Forest plots showed the 95% CI for the pooled point prevalence. Additionally, log odds ratios and their standard errors were utilized to find correlations between risk factors and unfavorable birth outcomes.

## Result

### Characteristics of the studies and study participants

A total of 65 cross-sectional studies published between 2008 and 2025 were included in this systematic review and meta-analysis, comprising 28,813 participants from 12 Sub-Saharan African countries that includes inclusion criteria. The largest number of studies came from Nigeria [21], followed by Ethiopia [14] and Ghana [10]. The lowest prevalence of primary dysmenorrhea was reported in Nigeria (41.0%), and the highest was reported in Ghana (97.2%). The factors included in this systematic review and meta-analysis were age (< 20 years), age at menarche (< 12 years), chocolate consumption, tea drinking, a family history of dysmenorrhea, irregular menstrual cycles, history of anxiety, irregular menstrual cycle, length of menstrual cycle (< 21 days), nulliparous, and sexual intercourse (Additional file 5).

### Study selection

**Fig. 1:**
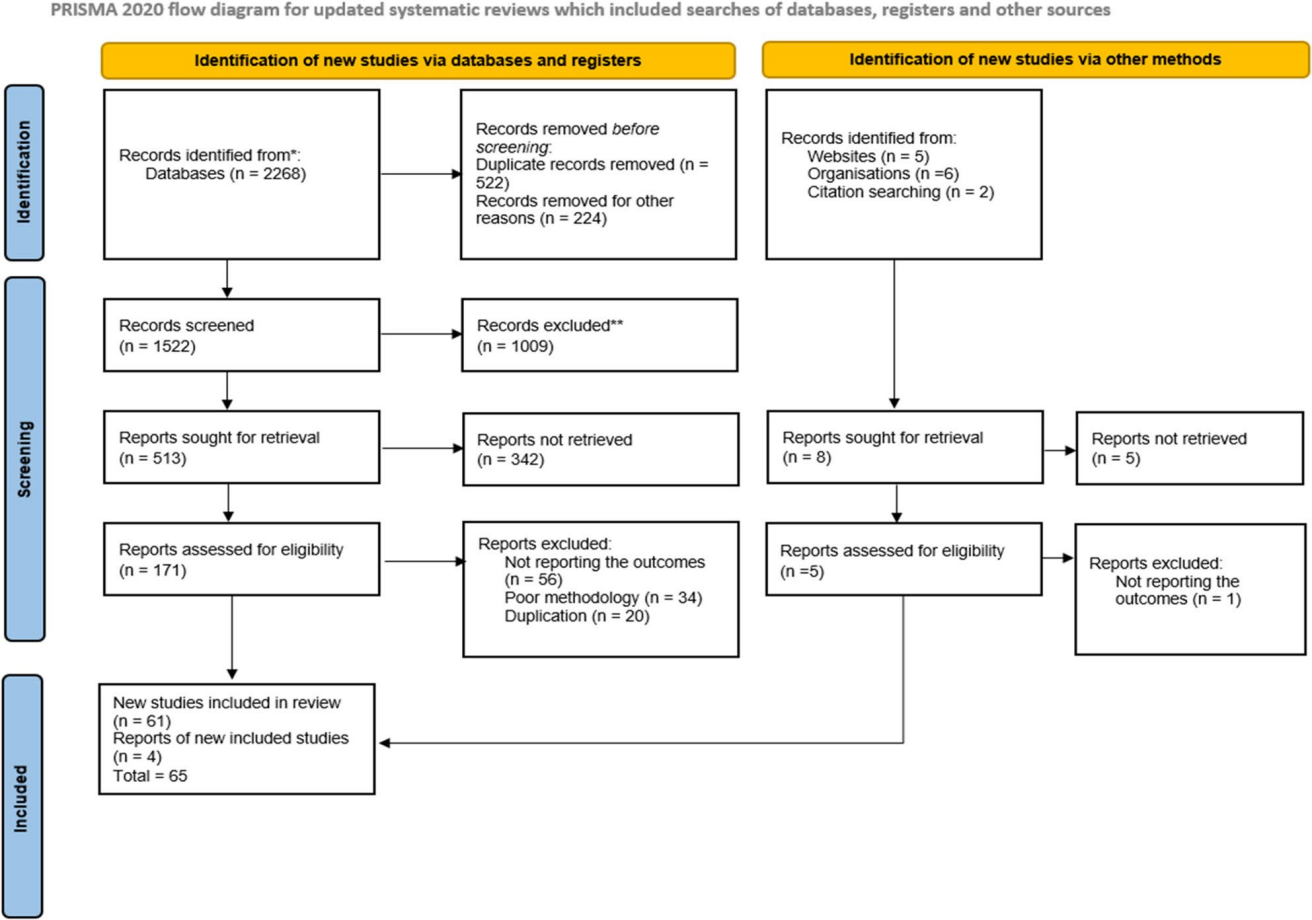
PRISMA 2020 Flow chart of prevalence and associated factors of primary dysmenorrhea among sub-Saharan Africa, 2025

The initial studies were located using pre-established search terms, filters across certain databases, and other relevant sources. After retrieval, duplicate entries were removed, and the records were imported into EndNote version 20 for reference management. The abstracts and titles of the remaining papers were independently screened by three authors using the pre-established inclusion criteria. Each study was now categorized as either included, excluded, or uncertain. For those deemed eligible or dubious, full-text articles were acquired and carefully assessed to determine their final eligibility (Fig. 1).

### The pooled prevalence of primary dysmenorrhea among women of sub-Saharan Africa

The forest plot summarizes the pooled prevalence estimate derived from 65 studies using a random-effects REML model. Individual study prevalence estimates show substantial variability, ranging widely across studies, with overlapping but heterogeneous confidence intervals. Despite this dispersion, most study estimates cluster around the overall pooled prevalence. The combined analysis yielded a pooled prevalence of 73.49% (95% CI: 70.95–76.03). Considerable heterogeneity was observed among the included studies (τ² = 103.95; I² = 96.43%; Q(64) = 2046.89, *p* < 0.001**)**, indicating that the variability in effect sizes was largely due to true differences between studies rather than chance alone. Nevertheless, under the random-effects model, all studies contributed relatively similar weights, and the pooled estimate provides a summary measure of prevalence across diverse study settings and populations (Table 1, Additional file 6).

**Table 1:** Pooled prevalence estimate and heterogeneity statistics from the random-effects meta-analysis

Outcome	Number of studies (*n*)	Total sample size	Pooled prevalence % (95% CI)	τ²	I² (%)	Q statistic (df)	*p*-value
Overall pooled prevalence	65	28,813	73.49 [70.95, 76.03]	103.95	96.43	2046.89 (64)	< 0.001

### Publication bias

**Fig. 2:**
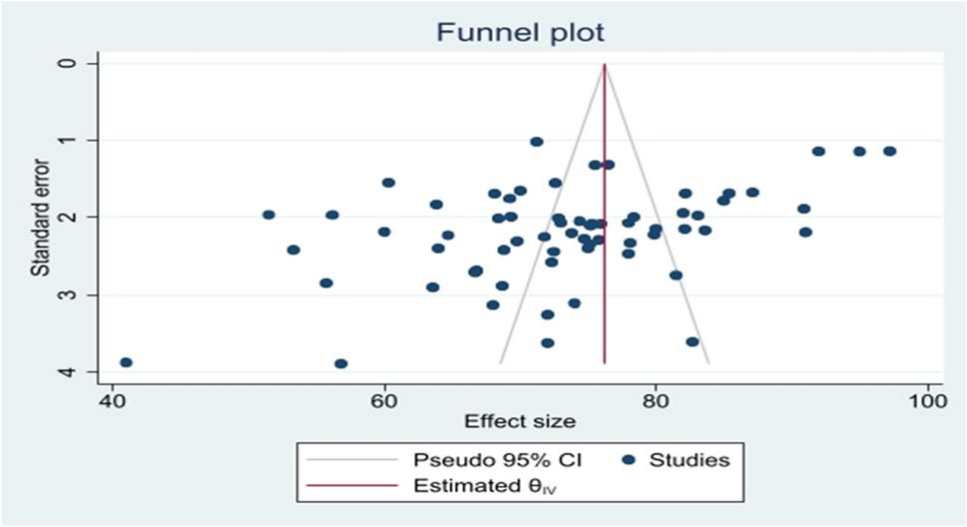
Funnel plot showing publication bias of the pooled prevalence of primary dysmenorrhea among sub-Saharan women, 2025

Publication bias was assessed visually using a funnel plot of effect size against standard error. The funnel plot demonstrated a relatively symmetrical distribution of the included studies around the pooled effect estimate. Most studies were located within the pseudo 95% confidence limits, with no clear evidence of asymmetry or a systematic absence of smaller studies on either side of the funnel. Although some dispersion was observed among studies with larger standard errors, this variability is consistent with expected sampling variation and underlying heterogeneity rather than selective publication (Fig. 2). Moreover, we assessed for heterogeneity by Egger’s regression test, and it showed that there was a publication bias (*p* = 0.0001). As a result, the nonparametric trim-and-fill analysis was conducted to assess the potential impact of publication bias on the pooled prevalence estimate. After applying the trim-and-fill procedure, the adjusted pooled estimate remained unchanged at 73.49% (95% CI: 70.96–76.03). The identical effect sizes and confidence intervals before and after adjustment indicate that publication bias had no detectable influence on the pooled prevalence estimate.

### The sensitivity analysis

Leave-one-out sensitivity analysis was performed to evaluate the influence of individual studies on the pooled prevalence estimate. Sequential omission of each study resulted in pooled prevalence estimates that varied only minimally, remaining tightly clustered around the overall estimate of approximately 73.49%, with all corresponding 95% CI largely overlapping. These findings indicate that no individual study disproportionately influenced the overall result, demonstrating the robustness and stability of the pooled prevalence estimate (Additional file 7).

### Subgroup analysis

Country-specific subgroup analysis revealed substantial variation in pooled prevalence across the included African countries. The highest estimates were reported in Côte d’Ivoire (79.9%), Zambia (78.0%), Ghana (77.1%), Nigeria (75.1%), Benin (75.3%), Uganda (74.9%), and Zimbabwe (75.9%), whereas lower estimates were noted in Cameroon (68.8%), Ethiopia (70.3%), Kenya (71.1%), Somalia (72.0%), and particularly Sudan (56.8%). Considerable within-country heterogeneity was evident in most countries with multiple studies, especially Cameroon, Ethiopia, Ghana, Nigeria, and Uganda, where I² values exceeded 90%, indicating substantial between-study variability. In contrast, Kenya showed no heterogeneity (I² = 0.0%), suggesting consistent findings across studies (Table 2, Additional file 8).

**Table 2:** The subgroup analysis of the pooled prevalence of primary dysmenorrhea among women of sub-Saharan Africa, 2025

Country	Number of studies	Pooled prevalence (95% CI)	τ²	I² (%)	Q(df)	*p*-value
Benin	2	75.34 [69.71, 80.97]	13.33	80.61	5.16 (1)	0.02
Cameroon	5	68.75 [61.32, 76.19]	69.64	97.24	101.11 (4)	< 0.001
Côte d’Ivoire	1	79.90 [75.55, 84.25]	—	—	—	—
Ethiopia	14	70.25 [65.86, 74.65]	66.06	94.15	239.12 (13)	< 0.001
Ghana	10	77.06 [69.39, 84.74]	148.38	97.40	416.55 (10)	< 0.001
Kenya	3	71.06 [67.73, 74.39]	0.00	0.00	1.37 (2)	0.50
Nigeria	22	75.06 [69.96, 80.16]	143.82	97.33	819.86 (21)	< 0.001
Somalia	1	72.00 [65.62, 78.38]	—	—	—	—
Sudan	1	56.80 [49.17, 64.43]	—	—	—	—
Uganda	4	74.89 [63.18, 86.61]	136.52	95.86	82.46 (3)	< 0.001
Zambia	1	78.00 73.94, 82.06]	—	—	—	—
Zimbabwe	1	75.90 [71.81, 79.99]	—	—	—	—
Overall	65	73.49 [70.95, 76.03]	103.95	96.43	2046.89 (64)	< 0.001

### Factors associated with the primary dysmenorrhea among women of sub-Saharan Africa

This meta-analysis found four key variables that were significantly associated with primary dysmenorrhea. Moreover, a random-effects model was performed for all significant variables (family history, irregular menstrual cycle, sexual intercourse, and length of menstrual cycle).

### Family History

**Fig. 3:**
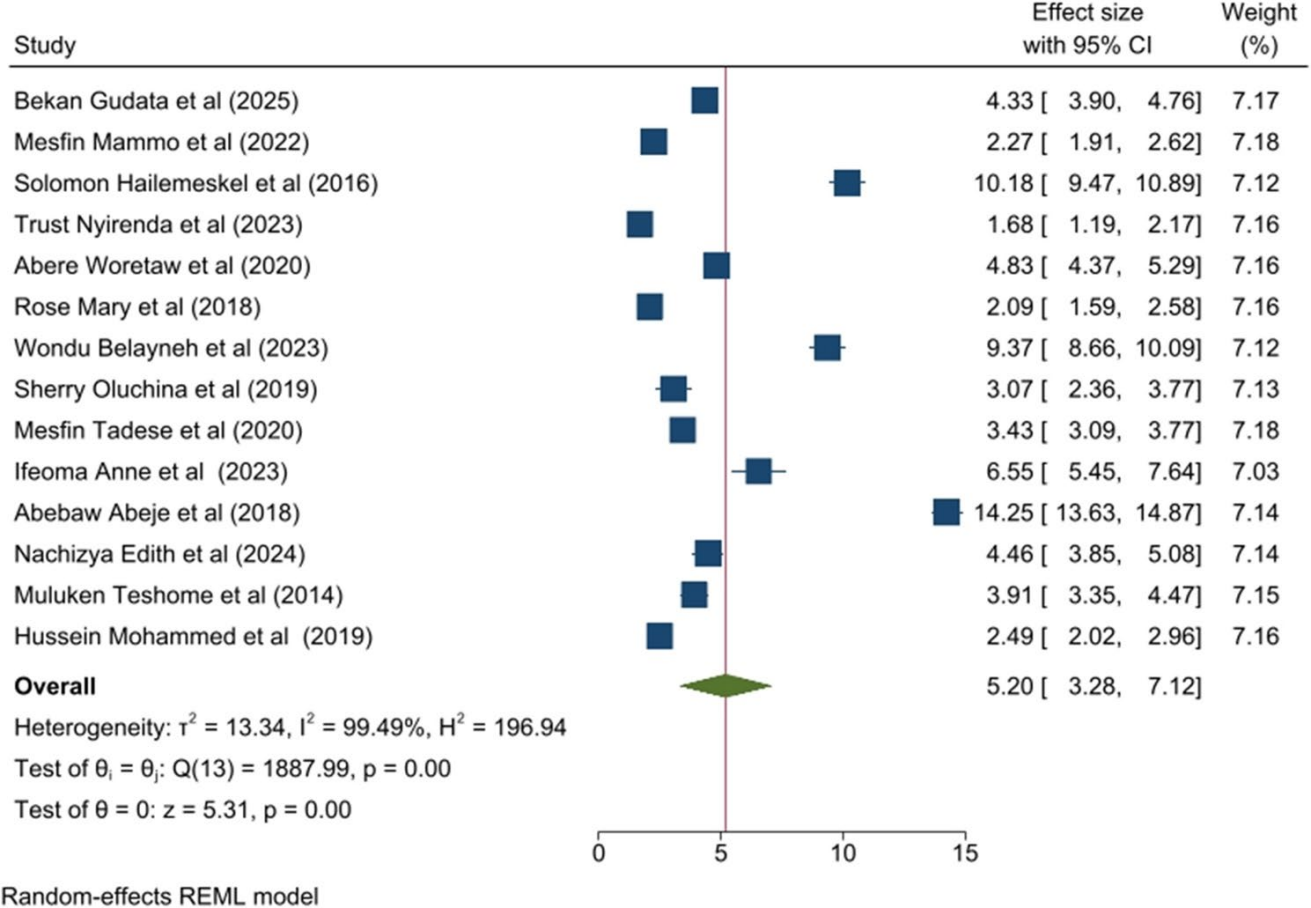
Forest plot of the pooled family history on primary dysmenorrhea among sub-Saharan Africa 2025

Eight of 12 included studies showed that a family history of dysmenorrhea was a strong predictor of dysmenorrhea among students in Ethiopia. The meta-analysis showed that family was significantly associated with PD among women of sub-Saharan Africa. Women who has family history of PD had 5.2 times higher odds of PD compared to those without a family history of PD (OR = 5.20; 95% CI: 3.28, 7.12), with no evidence of publication bias based on Egger’s test (*p* = 0.0870) (Fig. 3).

### Irregular menstrual cycle

**Fig. 4:**
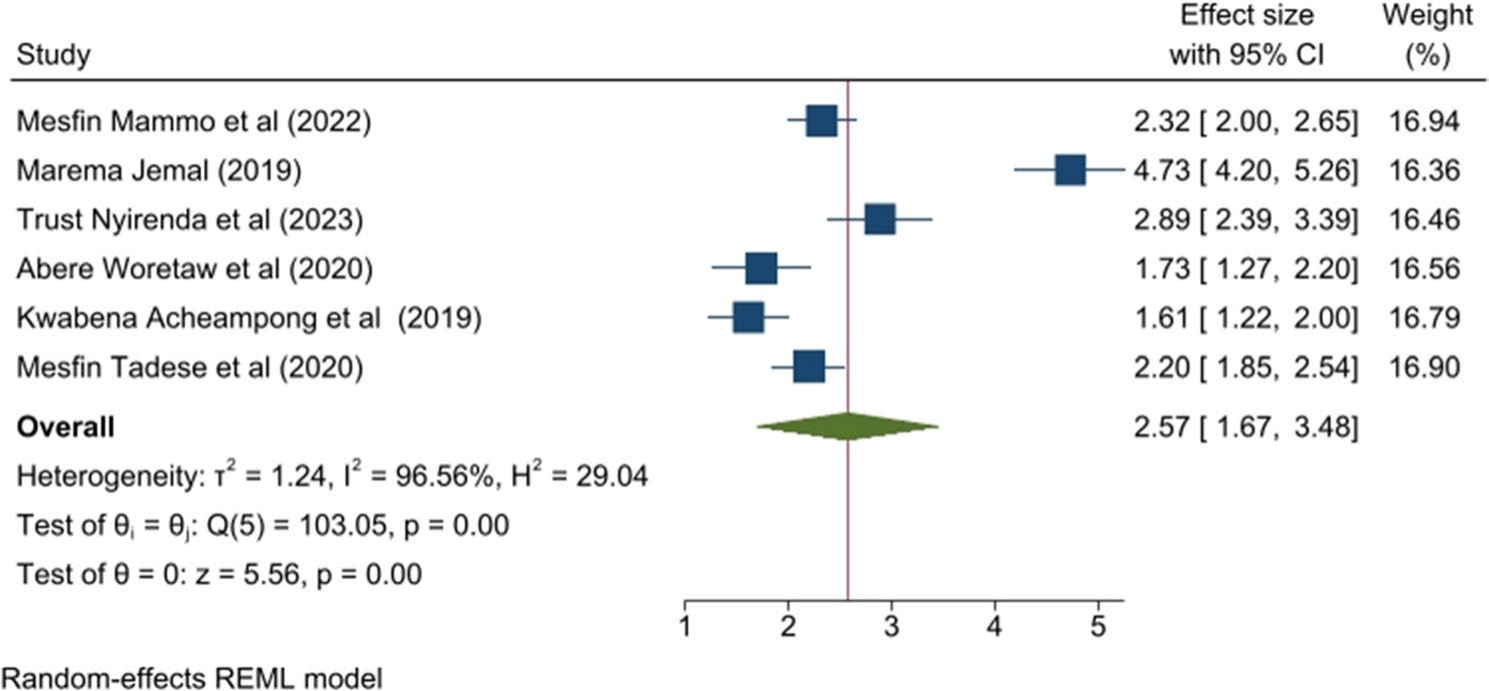
Forest plot of the pooled irregular menstrual cycle on primary dysmenorrhea among sub-Saharan Africa 2025

Women whose menstrual cycle was irregular had 2.57 times the odds of experiencing PD than women whose menstrual cycle was regular (OR = 2.57; 95% CI 1.67, 3.48). Moreover, the heterogeneity was high (I^2^ = 96.56%), and there was no evidence of publication bias by the Egger test (*p* = 0.1109) (Fig. 4).

### Length of menstrual cycle

**Fig. 5:**
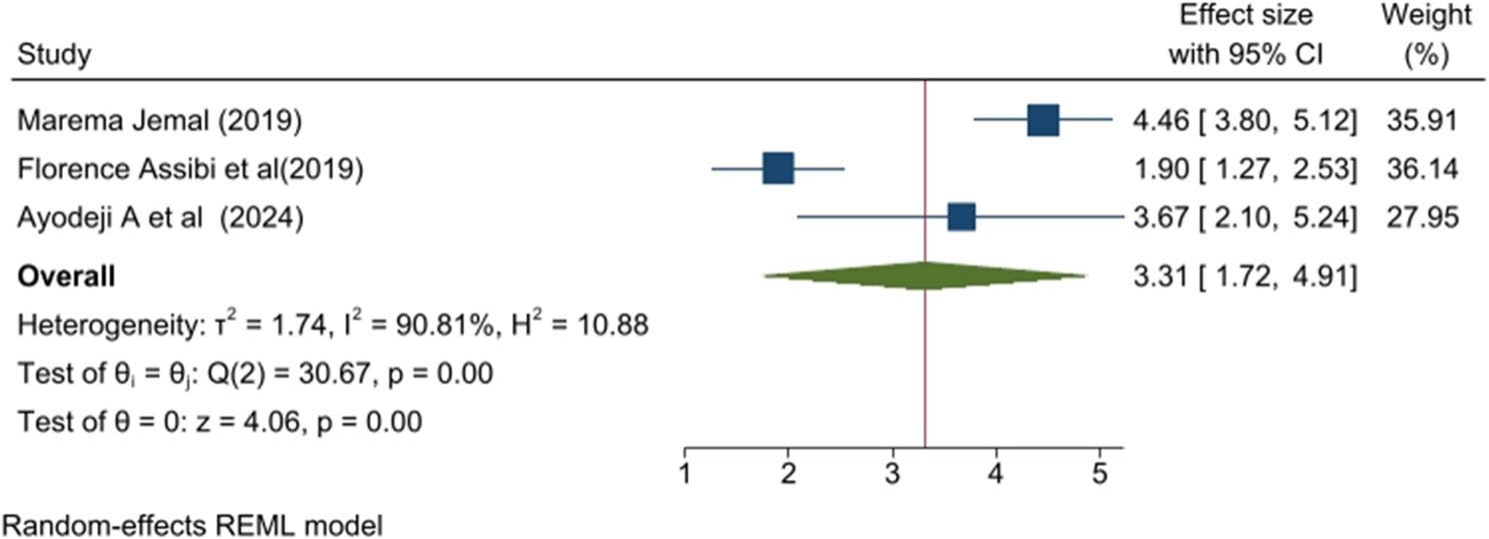
Forest plot of the pooled length of menstrual cycle on primary dysmenorrhea among sub-Saharan Africa 2025

Length of menstrual cycle was another variable that was strongly associated with PD. A woman whose menstrual cycle length was < 21 days was 3.31 times has higher risk of PD when compared with a woman whose menstrual cycle length was from 21 to 35 days. Additionally, the heterogeneity was high (I^2^ = 90.81%), with no publication bias by the Egger test (*p* = 0.8059) (Fig. 5).

### Sexual intercourse

**Fig. 6:**
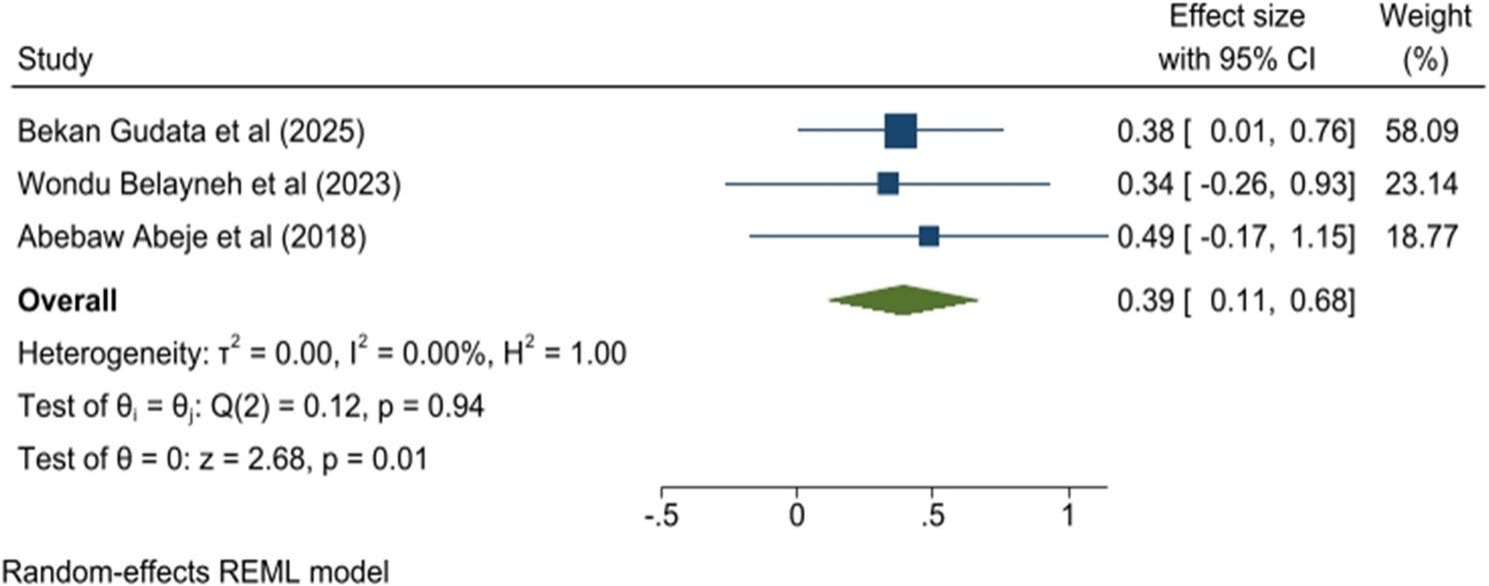
Forest plot of the pooled sexual intercourse on primary dysmenorrhea among sub-Saharan Africa 2025

The meta-analysis also identified that sexual intercourse was significantly associated with the prevalence of PD. A woman who started sexual intercourse has decreased the odds of having the prevalence of PD by 61% when compared with a woman who did not experience sexual intercourse (OR = 0.39; 95% CI 0.11, 0.68). Moreover, it did not show any heterogeneity (I^2^ = 0.00%), nor publication bias by the Egger test (*p* = 0.8995) (Fig. 6).

## Discussion

This systematic review and meta-analysis synthesizes available evidence on the prevalence and associated factors of primary dysmenorrhea among women in SSA. By pooling data from 65 cross-sectional studies conducted across multiple countries in the region, the review provides a comprehensive regional estimate of disease burden and identifies key factors associated with primary dysmenorrhea. The findings highlight primary dysmenorrhea as a common and significant reproductive health problem with important implications for women’s well-being, academic participation, and productivity.

The pooled prevalence of primary dysmenorrhea in Sub-Saharan Africa was 73.49%, indicating that nearly three-quarters of women experience menstrual pain. This estimate is comparable to pooled prevalence reported in recent global and regional systematic reviews and meta-analyses, though some variation exists across regions. A large global meta-analysis, which synthesized data from 70 countries, reported a prevalence 71.3% with higher pooled values observed among adolescents and young women [1]. Similarly, a systematic review and meta-analysis focusing on young women and students reported a pooled prevalence exceeding 71.1%, closely aligning with the estimate observed in the present study [6]. Moreover, a systematic review and meta-analysis from China has a pooled prevalence of primary dysmenorrhea as 70.3% [22]. This similarity likely reflects that most studies focused on adolescents and young women, who are more prone to primary dysmenorrhea due to higher prostaglandin activity and pain sensitivity [23]. Additionally, the use of cross-sectional designs, self-reported questionnaires, and school- or university-based populations in both SSA and global studies likely contributed to comparable prevalence estimates. However, the pooled prevalence of primary dysmenorrhea in SSA was lower than the pooled prevalence of dysmenorrhea in Central America (89.6%), Sri Lanka (97.7%) [1]. The possible reason may be the differences in study populations, cultural attitudes toward reporting menstrual pain, methodological variations, and age distributions. In particular, higher prevalence in these countries may be driven by studies focusing on adolescent girls in school-based settings, who often report more severe menstrual pain, whereas SSA studies included a broader age range and diverse educational settings, which could dilute prevalence estimates.

A family history of dysmenorrhea was the strongest predictor in this meta-analysis, with affected women having more than fivefold increased odds of experiencing menstrual pain. This finding is consistent with a systematic review and meta-analysis from Japan, which includes 77 studies, found that women with a family history of dysmenorrhea had significantly higher odds of the condition, highlighting that genetic predisposition and shared familial or behavioral factors contribute to dysmenorrhea risk in both developed and developing countries [24]. Moreover, a systematic review and meta-analysis from China with 23 studies among female college students found that family history of dysmenorrhea increased the odds of having primary dysmenorrhea [22]. This strong association may be due to genetic factors influencing uterine prostaglandin production, pain sensitivity, and hormonal regulation, as well as the intergenerational transmission of menstrual pain perceptions and coping behaviors [25].

Moreover, women with irregular menstrual cycle was also significantly more likely to experience primary dysmenorrhea. This finding was supported by a systematic review and meta-analysis from Japan [24]. A possible reason is that irregular cycles often reflect hormonal imbalances, particularly fluctuations in estrogen and progesterone, which can increase uterine prostaglandin production and uterine contractions, thereby intensifying menstrual pain [26]. Similarly, a short menstrual cycle length (< 21 days**)** was associated with increased odds of dysmenorrhea, possibly due to frequent ovulatory cycles and enhanced endometrial prostaglandin synthesis [26, 27].

In contrast, sexual intercourse was associated with a lower likelihood of primary dysmenorrhea. A possible reason is that sexual intercourse may stimulate the release of endorphins and oxytocin, which act as natural pain modulators and can increase pain tolerance and muscle relaxation, thereby potentially reducing menstrual pain. Endorphins released during sexual activity have been associated with increased pain thresholds and analgesic effects, and circulating oxytocin, which increases during sexual arousal and orgasm, has been linked to higher pain tolerance and modulation of pain responses [28].

### Strengths and limitations

This systematic review and meta-analysis have several strengths. It is the first comprehensive synthesis of evidence on the prevalence and associated factors of primary dysmenorrhea in Sub-Saharan Africa, incorporating data from 65 studies across 12 countries and a large combined sample size. The review followed PRISMA 2020 guidelines, was prospectively registered in PROSPERO, applied standardized JBI quality appraisal tools, and used robust random-effects meta-analytic methods with extensive sensitivity and subgroup analyses, enhancing the reliability of the findings.

However, all included studies were cross-sectional in design, which precludes causal inference between identified factors and primary dysmenorrhea. Substantial heterogeneity was observed across studies and country-specific subgroups, likely reflecting differences in study populations, measurement tools, age groups, and sociocultural contexts. In addition, the reliance on self-reported measures of dysmenorrhea may have introduced recall and reporting bias, potentially leading to over- or underestimation of prevalence.

## Conclusion

This systematic review and meta-analysis indicate that primary dysmenorrhea is highly prevalent in Sub-Saharan Africa, affecting nearly three-quarters of women. Family history of dysmenorrhea, irregular menstrual cycles, and short cycle length (< 21 days) were identified as significant risk factors, while sexual intercourse was associated with a lower likelihood of experiencing menstrual pain. These findings are consistent with global evidence and highlight the roles of family history, irregular menstrual cycle, short cycle length, and sexual intercourse in the occurrence of primary dysmenorrhea.

## Supplementary Information

Supplementary Material 1.

Supplementary Material 2.

Supplementary Material 3.

Supplementary Material 4.

Supplementary Material 5.

Supplementary Material 6.

Supplementary Material 7.

Supplementary Material 8.

## Data Availability

The data supporting the findings of this systematic review are available from the corresponding author upon reasonable request.

## Abbreviations

CI: Confidence Interval
CRD: Centre for Reviews and Dissemination
JBI: Joanna Briggs Institute
OR: Odds Ratio
PD: Primary Dysmenorrhea
PRISMA: Preferred Reporting Items for Systematic Reviews and Meta-Analyses
REML: Restricted Maximum Likelihood
SSA: Sub-Saharan Africa
