# Supplementary material for: Prevalence and associated factors of primary dysmenorrhea among women in sub-Saharan Africa: a systematic review and meta-analysis
Source: BMC Womens Health. 2026 Mar 5;26:198. doi: 10.1186/s12905-026-04379-1 (PMC13069715; doi:10.1186/s12905-026-04379-1)
Supplement: Supplementary file 2 — Supplementary Material 2. [file 12905_2026_4379_MOESM2_ESM.docx]

**Additional file 3: Results of JBI quality assessment for Prevalence Primary Dysmenorrhea among Young Women in Sub-Saharan Africa.**

The methodological quality of included studies was assessed using the Joanna Briggs Institute (JBI) critical appraisal tools. Studies reporting prevalence outcomes were appraised using the JBI checklist for prevalence studies, while studies reporting associated factors were appraised using the JBI analytical cross-sectional studies checklist

| Studies | Appropriate sampling frame | Appropriate sampling | Adequate sample size | Detailed setting description | Analysis with sufficient coverage | Valid Method to identify the condition | Reliable measurement | Appropriate statistical analysis | Adequate response rate | Total, out of 9 |
| --- | --- | --- | --- | --- | --- | --- | --- | --- | --- | --- |
| Mesfin Mammo(2022) | √ | √ | √ | √ | √ | √ | √ | √ | √ | 9 |
| Merema Jemal(2019) | √ | √ | × | √ | √ | √ | √ | √ | √ | 8 |
| Solomon Hailemeskel(2016) | √ | √ | √ | √ | √ | √ | √ | √ | √ | 9 |
| Trust Nyirenda(2023) | √ | √ | √ | √ | √ | √ | √ | √ | √ | 9 |
| Mesfin Tadese(2020) | √ | √ | √ | √ | √ | √ | √ | √ | √ | 9 |
| Humphrey Beja(2024) | √ | √ | × | √ | √ | √ | √ | √ | √ | 8 |
| Abera Woretaw (2020) | √ | √ | √ | √ | √ | √ | √ | √ | √ | 9 |
| A. TITILAYO(2009) | √ | √ | √ | √ | × | × | √ | √ | √ | 7 |
| Bekan Gudata(2025) | √ | √ | √ | √ | √ | √ | √ | √ | √ | 9 |
| Mahublo Vinadou(2020) | √ | √ | √ | √ | √ | √ | √ | × | √ | 8 |
| Sidi I(2016) | √ | √ | √ | √ | √ | √ | √ | √ | √ | 9 |
| Nachizya Edith (2024) | √ | √ | √ | √ | √ | √ | √ | √ | √ | 9 |
| Jim Amisi(2024) | √ | √ | √ | √ | √ | √ | √ | √ | × | 8 |
| Abdikadir Ahmed(2021) | √ | UC | × | × | × | UC | × | × | UC | 1 |
| Aribo Ekpe(2024) | √ | √ | √ | √ | √ | √ | √ | √ | √ | 9 |
| Umeobieri Ancilla(2022) | √ | √ | × | √ | √ | √ | √ | √ | × | 7 |
| Axel Mbvoumi(2020) | √ | √ | √ | √ | √ | √ | √ | √ | × | 8 |
| Faustina Chiamaka(2023) | √ | √ | √ | √ | √ | √ | √ | √ | UC | 8 |
| Henry Nwude(2025) | √ | √ | × | √ | √ | √ | UC | √ | × | 6 |
| Deborah Tolulope(2024) | √ | √ | √ | √ | √ | √ | √ | √ | √ | 9 |
| Jimoh Mohammed(2018) | √ | √ | √ | √ | √ | √ | √ | √ | √ | 9 |
| Olabisi M(2008) | √ | √ | √ | √ | √ | √ | √ | √ | √ | 9 |
| Florence Assibi(2019) | √ | √ | √ | √ | √ | √ | √ | √ | √ | 9 |
| NAKAME ROSE (2015) |  |  |  |  |  |  |  |  |  |  |
| Wondu Belayneh(2023) | √ | √ | √ | √ | √ | √ | √ | √ | √ | 9 |
| Rose Mary (2018) | √ | × | √ | × | √ | √ | √ | √ | √ | 7 |
| Kwabena Acheampong(2019) | √ | × | √ | √ | √ | √ | √ | √ | √ | 8 |
| Gumanga S K(2012) | √ | √ | √ | √ | √ | √ | √ | √ | √ | 9 |
| Nwogbo(2024) | √ | √ | √ | √ | √ | √ | √ | √ | √ | 9 |
| Evans Paul Kwame (2018) | √ | √ | √ | √ | √ | √ | √ | √ | √ | 9 |
| Tenagnee, Kebed(2019) | √ | √ | √ | √ | × | × | × | × | × | 4 |
| Mboua Batoum(2023) | √ | √ | √ | √ | √ | √ | √ | √ | √ | 9 |
| Adjoa Enyidado (2024) | √ | √ | × | √ | √ | √ | √ | √ | UC | 7 |
| Folasade Adenike (2017) | √ | √ | √ | √ | √ | √ | √ | √ | × | 8 |
| Gabriel G. Akunna(2020) | √ | √ | √ | √ | √ | √ | √ | √ | √ | 9 |
| Ayodeji Adeyemo(2021) | √ | √ | √ | √ | √ | √ | √ | √ | √ | 9 |
| AdekunbiFarotimi(2015) | √ | √ | √ | √ | √ | √ | √ | √ | √ | 9 |
| Ezebialu IU(2021) | √ | √ | √ | √ | √ | √ | √ | √ | √ | 9 |
| Oluwole EO(2020) | √ | √ | √ | √ | √ | √ | √ | √ | √ | 9 |
| Hasford KE(2023) | √ | × | × | √ | √ | √ | √ | √ | √ | 7 |
| Bayor Surazu(2025) | √ | √ | √ | √ | √ | √ | √ | √ | √ | 9 |
| Abebaw Abeje (2018) | √ | √ | √ | √ | √ | √ | √ | √ | √ | 9 |
| Hussein Mohammed (2019) | √ | √ | √ | √ | √ | √ | √ | √ | √ | 9 |
| Derseh BT(2017) | √ | √ | √ | √ | √ | √ | √ | √ | √ | 9 |
| Sherry Oluchina (2025) | √ | √ | √ | √ | √ | × | √ | √ | √ | 8 |
| Alex Kagia(2016 | √ | √ | √ | √ | × | √ | × | √ | √ | 7 |
| Eseza Teopistar (2024) | √ | × | √ | √ | √ | √ | √ | √ | √ | 8 |
| Emmanuel Odongo(2023) | **√** | **√** | **√** | **√** | **√** | **√** | **√** | **√** | **√** | 9 |
| Jeanne Hortence (2019) | **√** | **√** | **√** | **√** | **√** | **√** | **√** | **√** | **√** | 9 |
| Ongbayokolak Na(2020) | **√** | UC | UC | **√** | **√** | **√** | **√** | **√** | UC | 6 |
| Michèle Florence (2025) | **√** | **√** | **√** | **√** | **√** | **√** | **√** | **√** | **√** | 9 |
| Abubakari Wuni (2023) | **√** | **√** | **√** | **√** | **√** | **√** | **√** | **√** | **√** | 9 |
| Anthony Ike Wegbom(2025) | **√** | **√** | **√** | **√** | **√** | **√** | **√** | **√** | **√** | 9 |
| Ayokunle Osonuga(2019) | **√** | × | **√** | **√** | **√** | **√** | **√** | **√** | × | 7 |
| Comfort Emma (2024) | **√** | **√** | **√** | **√** | **√** | **√** | **√** | **√** | **√** | 9 |
| Damilola M(2019) | **√** | **√** | **√** | **√** | **√** | **√** | **√** | **√** | **√** | 9 |
| Edith C(2021) | **√** | **√** | **√** | **√** | **√** | **√** | **√** | **√** | **√** | 9 |
| Ifeoma Anne (2023) | **√** | **√** | × | **√** | **√** | **√** | **√** | **√** | × | 7 |
| Ogunyemi(2022) | **√** | **√** | **√** | **√** | **√** | **√** | **√** | **√** | **√** | 9 |
| Tiruye Tilahun M(2022) | **√** | **√** | **√** | **√** | **√** | **√** | **√** | **√** | **√** | 9 |
| Waliu Babatunde (2024) | **√** | **√** | × | **√** | **√** | **√** | **√** | **√** | × | 7 |
| Yasir Salih(2025) | **√** | **√** | × | **√** | **√** | **√** | **√** | **√** | × | 7 |
| Muluken Teshome (2014) | **√** | **√** | **√** | **√** | **√** | **√** | **√** | **√** | **√** | 9 |
| Prisca N’Gatta(2023) | **√** | **√** | **√** | **√** | **√** | **√** | **√** | **√** | × | 8 |
| Olutunde Ademola (2016) | **√** | **√** | **√** | **√** | **√** | **√** | **√** | **√** | × | 8 |
| Munewar Usman et al (2025) | **√** | **√** | **√** | **√** | **√** | **√** | **√** | **√** | **√** | 9 |
