# Supplementary material for: Prevalence and associated factors of primary dysmenorrhea among women in sub-Saharan Africa: a systematic review and meta-analysis
Source: BMC Womens Health. 2026 Mar 5;26:198. doi: 10.1186/s12905-026-04379-1 (PMC13069715; doi:10.1186/s12905-026-04379-1)
Supplement: Supplementary file 3 — Supplementary Material 3. [file 12905_2026_4379_MOESM3_ESM.docx]

| Studies | Clear inclusion criteria | Detailed study subjects and setting | The exposure was measured in a valid and reliable | Objective, standard criteria used for the measurement of the condition | Confounding factors identified | Strategies to deal with confounding factors stated | The outcomes are measured in a valid and reliable manner | An appropriate statistical analysis was used | Total |
| --- | --- | --- | --- | --- | --- | --- | --- | --- | --- |
| Evans Paul et al (2018) | √ | √ | √ | √ | √ | √ | UC | √ | 7 |
| Sherry Oluchina (2019) | √ | √ | √ | √ | √ | √ | √ | √ | 8 |
| Mesfin Tadese et al (2020) | √ | √ | √ | √ | √ | √ | √ | √ | 8 |
| Ayodeji A et al (2024) | √ | √ | √ | √ | √ | √ | UC | √ | 7 |
| Ifeoma Anne (2023) | √ | √ | √ | √ | UC | UC | √ | √ | 6 |
| Abebaw Abeje(2018) | √ | √ | √ | √ | √ | √ | √ | √ | 8 |
| Nachizya Edith (2024) | √ | √ | √ | √ | √ | √ | √ | √ | 8 |
| Bekan (2025) | √ | √ | √ | √ | √ | √ | √ | √ | 8 |
| Mesfin Mammo(2022) | √ | √ | √ | √ | √ | √ | √ | √ | 8 |
| Marema (2019) | √ | √ | √ | √ | √ | UC | √ | √ | 7 |
| Solomon Hailemeskel (2016) | √ | √ | √ | √ | √ | √ | √ | √ | 8 |
| Trust Nyirenda (2023) | √ | √ | √ | √ | √ | UC | √ | √ | 7 |
| Abere Woretaw(2020) | √ | √ | √ | √ | √ | √ | √ | √ | 8 |
| Florence Assibi Ziba(2019) | √ | √ | √ | √ | √ | √ | √ | √ | 8 |
| Rose Mary Nakame (2018) | √ | √ | √ | √ | √ | UC | √ | √ | 7 |
| Mahublo Vinadou (2020) | √ | √ | √ | √ | √ | UC | √ | √ | 7 |
| Wondu Belayneh (2023) | √ | √ | √ | √ | √ | √ | √ | √ | 8 |
| Kwabena Acheampong (2019) | √ | √ | √ | √ | √ | √ | √ | √ | 8 |
| Ibrahim Ologele(2021) | √ | √ | UC | √ | UC | UC | UC | UC | 3 |
| Oluwabunmi Victoria | √ | √ | UC | √ | UC | UC | UC | UC | 3 |
| Ayokunle Osonuga | √ | √ | √ | × | × | × | × | × | 3 |
| Munewar Usman et al (2025) | √ | √ | √ | √ | √ | √ | √ | √ | 8 |
