# Supplementary material for: Prevalence and associated factors of primary dysmenorrhea among women in sub-Saharan Africa: a systematic review and meta-analysis
Source: BMC Womens Health. 2026 Mar 5;26:198. doi: 10.1186/s12905-026-04379-1 (PMC13069715; doi:10.1186/s12905-026-04379-1)
Supplement: Supplementary file 4 — Supplementary Material 4. [file 12905_2026_4379_MOESM4_ESM.docx]

| Authors | Country | Study Design | Sample size | Prevalence | Outcome variables | Risk of Bias |
| --- | --- | --- | --- | --- | --- | --- |
| Bekan Gudata (2025) | Ethiopia | Cross-sectional | 532 | 68. | Sexual intercourse, drinking tea, History of anxiety, and Family history | Low |
| Mesfin Mammo(2022) | Ethiopia | Cross-sectional | 773 | 70 | Age (<20 years), Irregular menstrual cycle, Family history | Low |
| Marema (2019) | Ethiopia | Cross-sectional | 340 | 75.1 | Irregular menstrual cycle, Length of menstrual cycle (<21 days) | Low |
| Solomon Hailemeskel (2016) | Ethiopia | Cross-sectional | 440 | 85.4 | History of anxiety, Drinking tea, Nulliparous, Family history | Low |
| Trust Nyirenda (2023) | Zimbabwe | Cross-sectional | 420 | 75.9 | Family history, Age at menarche (<12)  Irregular menstrual cycle, Nulliparous | Low |
| Abere Woretaw Azagew (2020) | Ethiopia | Cross-sectional | 459 | 64.7 | Irregular menstrual cycle  Family history | Low |
| Florence Assibi (2019) | Ghana | Cross-sectional | 400 | 85 | Age at menarche (<12)  Length of menstrual cycle (<21 days) | Low |
| Rose Mary Nakame (2018) | Uganda | Cross-sectional | 351 | 75.8 | Family history | Low |
| Mahublo Vinadou (2020) | Benin | Cross-sectional | 822 | 72.6 | Age (>20 years), Nulliparous | Low |
| Wondu Belayneh (2023) | Ethiopia | Cross-sectional | 348 | 80 | Sexual intercourse  Family history  History of anxiety | Low |
| Kwabena Acheampong (2019) | Ghana | Cross-sectional | 760 | 68.1 | Irregular menstrual cycle | Low |
| Evans Paul (2018) | Ghana | Cross-sectional | 293 | 83.6 | Age (<20 years) | Low |
| Sherry Oluchina | Kenya | Cross-sectional | 222 | 68 | Family history | Low |
| Mesfin Tadese (2020) | Ethiopia | Cross-sectional | 647 | 51.5 | Irregular menstrual cycle  Family history | Low |
| Ayodeji A (2024) | Nigeria | Cross-sectional | 319 | 82.1 | Length of menstrual cycle (<21 days) | Low |
| Anthony Ike (2025) | Nigeria | Cross-sectional | 564 | 92 | Nulliparous | Low |
| Ifeoma Anne (2023) | Nigeria | Cross-sectional | 110 | 82.7 | Family history | Low |
| Abebaw Abeje (2018) | Ethiopia | Cross-sectional | 539 | 69.3 | Family history, Drinking tea  Age at menarche (<12), Sexual intercourse | Low |
| Nachizya Edith (2024) | Zambia | Cross-sectional | 400 | 78 | Family history | Low |
| Muluken Teshome (2014) | Ethiopia | Cross-sectional | 491 | 72.8 | Family history | Low |
| Humphrey Beja(2024) | Uganda | Cross-sectional | 232 | 90.9 | **_** | Low |
| A. TITILAYO(2009) | Nigeria | Cross-sectional | 400 | 64 | **_** | Low |
| Sidi I(2016) | Benin | Cross-sectional | 425 | 78.35 | **_** | Low |
| Jim Amisi(2024) | Kenya | Cross-sectional | 334 | 72.5 | **_** | Low |
| Abdikadir Ahmed (2021) | Somalia | Cross-sectional | 190 | 72 | **_** | Low |
| Aribo Ekpe (2024) | Nigeria | Cross-sectional | 1000 | 60.3 | **_** | Low |
| Umeobieri Ancilla (2022) | Nigeria | Cross-sectional | 390 | 82 | **_** | Low |
| Axel Mbvoumi (2020) | Cameroon | Cross-sectional | 637 | 56.2 | **_** | Low |
| Faustina Chiamaka(2023) | Nigeria | Cross-sectional | 200 | 81.5 | **_** | Low |
| Henry Nwude(2025) | Nigeria | Cross-sectional | 161 | 41 | **_** | Low |
| Deborah Tolulope(2024) | Nigeria | Cross-sectional | 397 | 69.8 | **_** | Low |
| Jimoh-Mohammed(2018) | Nigeria | Cross-sectional | 400 | 71.8 | **_** | Low |
| Olabisi M(2008) | Nigeria | Cross-sectional | 424 | 53.3 | **_** | Low |
| Gumanga S K(2012) | Ghana | Cross-sectional | 453 | 74.4 | **_** | Low |
| Nwogbo(2024) | Nigeria | Cross-sectional | 362 | 95 | **_** | Low |
| Tenagnee, Kebed(2019) | Ethiopia | Cross-sectional | 430 | 75.3 | **_** | Low |
| Mboua Batoum(2023) | Cameroon | Cross-sectional | 1978 | 71.2 | **_** | Low |
| Adjoa Enyida (2024) | Ghana | Cross-sectional | 211 | 97.2 | **_** | Low |
| Folasade Adenike (2017) | Nigeria | Cross-sectional | 360 | 83.1 | **_** | Low |
| Gabriel G. (2020) | Nigeria | Cross-sectional | 400 | 87.1 | **_** | Low |
| Adekunbi A Farotimi(2015) | Nigeria | Cross-sectional | 315 | 78.1 | **_** | Low |
| Ezebialu IU(2021) | Nigeria | Cross-sectional | 516 | 82.2 | **_** | Low |
| Oluwole EO(2020) | Nigeria | Cross-sectional | 420 | 75.2 | **_** | Low |
| Hasford KE(2023) | Ghana | Cross-sectional | 170 | 91 | **_** | Low |
| Bayor Surazu(2025) | Ghana | Cross-sectional | 305 | 55.7 | **_** | Low |
| Hussein Mohammed (2019) | Ethiopia | Cross-sectional | 693 | 69.26 | \| Family history \| \| --- \| \| Age at menarche (<12) \| | Low |
| Derseh BT(2017) | Ethiopia | Cross-sectional | 307 | 66.8 | **_** | Low |
| Munewar Usman et al (2025) | Ethiopia | Cross-sectional | 503 | 60.0 | Family history  History of anxiety  Irregular menstrual cycle | Low |
| Alex Kagia(2016 | Kenya | Cross-sectional | 154 | 72 | **_** | Low |
| Eseza Teopistar (2024) | Uganda | Cross-sectional | 259 | 68.7 | **_** | Low |
| Emmanuel Odongo(2023) | Uganda | Cross-sectional | 275 | 63.6 | **_** | Low |
| Jeanne Hortence (2019) | Cameroon | Cross-sectional | 1059 | 75.5 | **_** | Low |
| Ongbayokolak (2020) | Cameroon | Cross-sectional | 689 | 63.86 | **_** | Low |
| Michèle Florence (2025) | Cameroon | Cross-sectional | 1045 | 76.5 | **_** | Low |
| Abubakari Wuni (2023) | Ghana | Cross-sectional | 303 | 66.7 | **_** | Low |
| Ayokunle (2019) | Ghana | Cross-sectional | 200 | 74 | **_** | Low |
| Comfort Emma (2024) | Ghana | Cross-sectional | 400 | 73.8 | **_** | Low |
| Damilola M(2019) | Nigeria | Cross-sectional | 460 | 73 | **_** | Low |
| Edith C(2021) | Nigeria | Cross-sectional | 326 | 75 | **_** | Low |
| Ogunyemi(2022) | Nigeria | Cross-sectional | 365 | 68.8 | **_** | Low |
| Tiruye Tilahun Mesele(2022) | Ethiopia | Cross-sectional | 365 | 74.7 | **_** | Low |
| Waliu Babatunde (2024) | Nigeria | Cross-sectional | 300 | 72.33 | **_** | Low |
| Yasir Salih(2025) | Sudan | Cross-sectional | 162 | 56.8 | **_** | Low |
| Prisca N’Gatta(2023) | Cote d’Ivoire | Cross-sectional | 326 | 79.9 | **_** | Low |
| Olutunde Ademola (2016) | Nigeria | Cross-sectional | 282 | 78 | **_** | Low |
