# Supplementary figures and images for: Prevalence and associated factors of primary dysmenorrhea among women in sub-Saharan Africa: a systematic review and meta-analysis
Source: BMC Womens Health. 2026 Mar 5;26:198. doi: 10.1186/s12905-026-04379-1 (PMC13069715; doi:10.1186/s12905-026-04379-1)

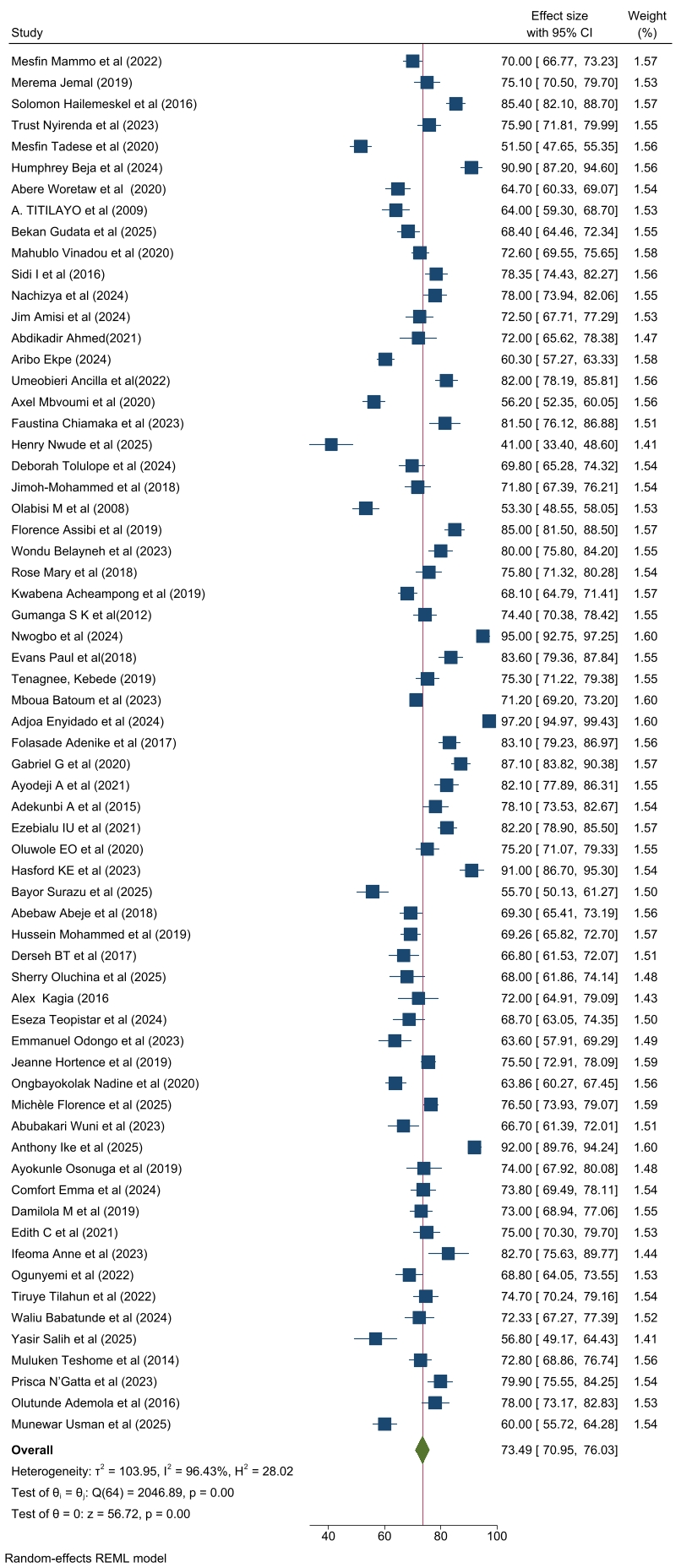

Supplement: Supplementary file 5 — Supplementary Material 5. [file 12905_2026_4379_MOESM5_ESM.docx]

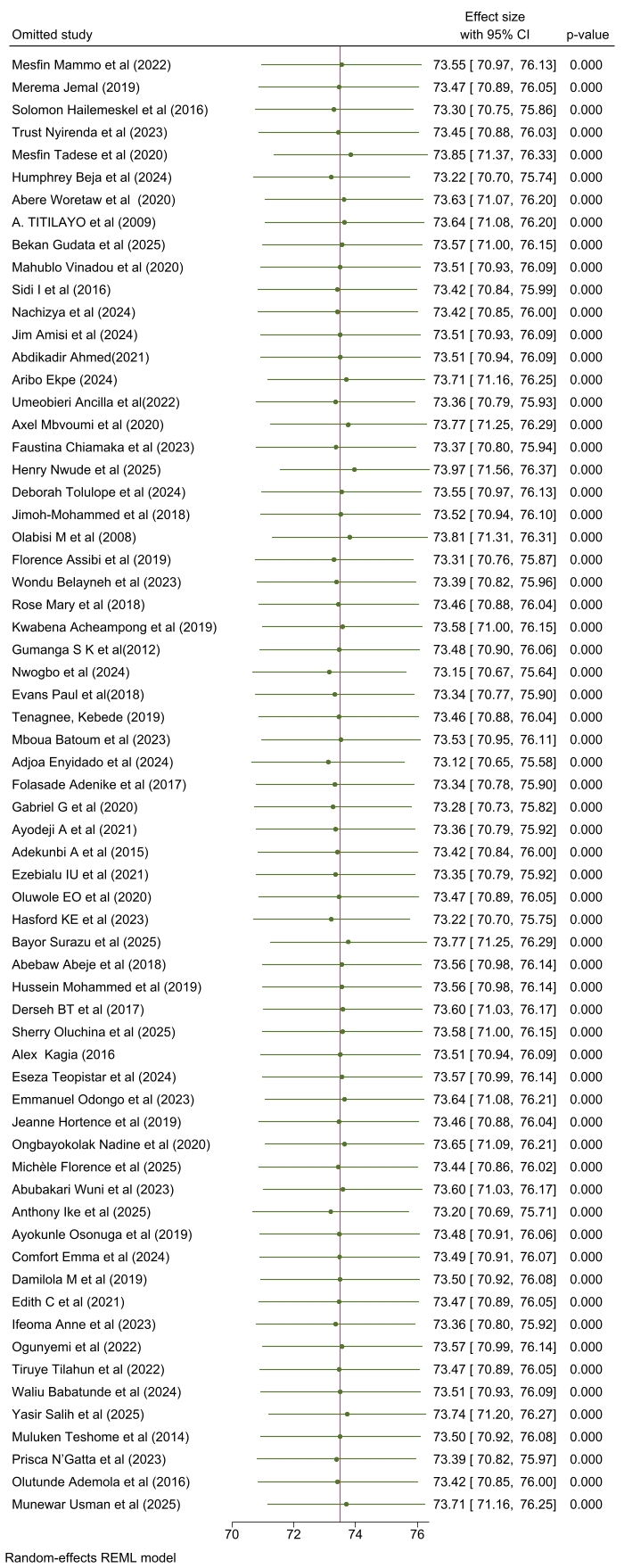

Supplement: Supplementary file 6 — Supplementary Material 6. [file 12905_2026_4379_MOESM6_ESM.docx]

**
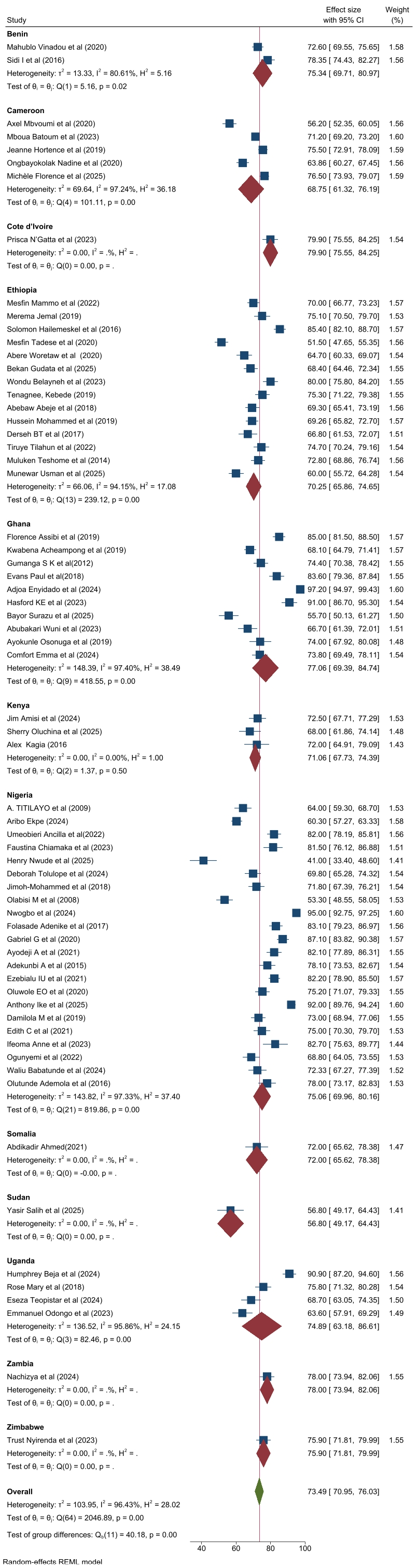
**

Supplement: Supplementary file 7 — Supplementary Material 7. [file 12905_2026_4379_MOESM7_ESM.docx]
