# Supplementary material for: Prevalence and associated factors of primary dysmenorrhea among women in sub-Saharan Africa: a systematic review and meta-analysis
Source: BMC Womens Health. 2026 Mar 5;26:198. doi: 10.1186/s12905-026-04379-1 (PMC13069715; doi:10.1186/s12905-026-04379-1)
Supplement: Supplementary file 8 — Supplementary Material 8. [file 12905_2026_4379_MOESM8_ESM.docx]

| Databases | Searching terms | Number of studies |
| --- | --- | --- |
| PubMed | *(“Dysmenorrhea” OR “primary dysmenorrhea” OR “menstrual pain”) AND (“Prevalence” OR “epidemiology” OR “magnitude” OR “frequency” OR “incidence”) AND (“Ethiopia” OR “Burundi” OR “Comoros” OR “Djibouti” OR “Eritrea” OR “Kenya” OR “Madagascar” OR “Malawi” OR “Mauritius” OR “Mozambique” OR “Rwanda” OR “Seychelles” OR “Somalia” OR “South Sudan” OR “Tanzania” OR “Uganda” OR “Zambia” OR “Zimbabwe” OR “Benin” OR “Burkina Faso” OR “Cabo Verde” OR “Côte d’Ivoire” OR “The Gambia” OR “Ghana” OR “Guinea” OR “Guinea-Bissau” OR “Liberia” OR “Mali” OR “Niger” OR “Nigeria” OR “Senegal” OR “Sierra Leone” OR “Togo” OR “Angola” OR “Cameroon” OR “Central African Republic” OR “Chad” OR “Republic of the Congo” OR “Democratic Republic of the Congo” OR “Equatorial Guinea” OR “Gabon” OR “São Tomé and Príncipe” OR “Botswana” OR “Eswatini” OR “Lesotho” OR “Namibia” OR “South Africa”)* | 115 |
| Google scholar | Prevalence and Associated Factors of Primary Dysmenorrhea among Young Women in Sub-Saharan Africa | **1550** |
| HINARI | Prevalence and Associated Factors of Primary Dysmenorrhea among Young Women in Sub-Saharan Africa | 368 |
| Scopus | (Dysmenorrhea) OR (primary dysmenorrhea)) OR (menstrual pain)) AND (Prevalence)) OR (epidemiology)) OR (magnitude)) OR (frequency)) OR (incidence)) AND (Risk Factors)) OR (associated factor)) OR (determinant)) OR (predictor)) AND (Sub-Saharan Africa)) | 128 |
| Other data sources | Google, MEDLINE, Cochrane Library, and Web of Science | 94 |
| Other methods | Manual searching, gray searching, Forward citation tracking, and university repositories | 13 |
| Total |  | 2268 |
